# Intrinsic motivation for singing in songbirds is enhanced by temporary singing suppression and regulated by dopamine

**DOI:** 10.1038/s41598-021-99456-w

**Published:** 2021-10-13

**Authors:** Yunbok Kim, Sojeong Kwon, Raghav Rajan, Chihiro Mori, Satoshi Kojima

**Affiliations:** 1 Sensory and Motor Systems Research Group, Korea Brain Research Institute, 61, Cheomdan-ro, Dong-gu, Daegu, 41068 South Korea; 2 Division of Biology, Indian Institute of Science Education and Research Pune, Pune, Maharashtra India; 3 Department of Molecular Biology, Faculty of Pharmaceutical Sciences, Teikyo University, Tokyo, Japan

**Keywords:** Neuroscience, Psychology, Zoology

## Abstract

Behaviors driven by intrinsic motivation are critical for development and optimization of physical and brain functions, but their underlying mechanisms are not well studied due to the complexity and autonomy of the behavior. Songbirds, such as zebra finches, offer a unique opportunity to study neural substrates of intrinsic motivation because they spontaneously produce many renditions of songs with highly-quantifiable structure for vocal practice, even in the absence of apparent recipients (“undirected singing”). Neural substrates underlying intrinsic motivation for undirected singing are still poorly understood partly because singing motivation cannot be easily manipulated due to its autonomy. Also, undirected singing itself acts as an internal reward, which could increase singing motivation, leading to difficulty in measuring singing motivation independent of singing-associated reward. Here, we report a simple procedure to easily manipulate and quantify intrinsic motivation for undirected singing independent of singing-associated reward. We demonstrate that intrinsic motivation for undirected singing is dramatically enhanced by temporary suppression of singing behavior and the degree of enhancement depends on the duration of suppression. Moreover, by examining latencies to the first song following singing suppression as a measure of singing motivation independent of singing-associated reward, we demonstrate that intrinsic singing motivation is critically regulated by dopamine through D2 receptors. These results provide a simple experimental tool to manipulate and measure the intrinsic motivation for undirected singing and illustrate the importance of zebra finches as a model system to study the neural basis of intrinsically-motivated behaviors.

## Introduction

Humans spontaneously engage in various activities, such as playing puzzles and sports, even without receiving any immediate external reward such as money or awards. These behaviors are driven by intrinsic motivation, which arises within individuals for internal satisfaction, as opposed to extrinsic motivation, which involves engaging in a behavior to gain external rewards or avoid punishment. Intrinsic motivation is critical not only for acquiring skills and knowledge but also for the development and optimization of cognitive, social, and physical functions throughout life^1,2^. Recently, empirical studies of neural substrates underlying intrinsic motivation have begun, but are limited mainly to non-invasive approaches in humans^3,4^. One major obstacle in understanding the neural mechanisms of intrinsic motivation at the circuit and cellular level is the lack of a suitable animal model.

**Figure 1:**
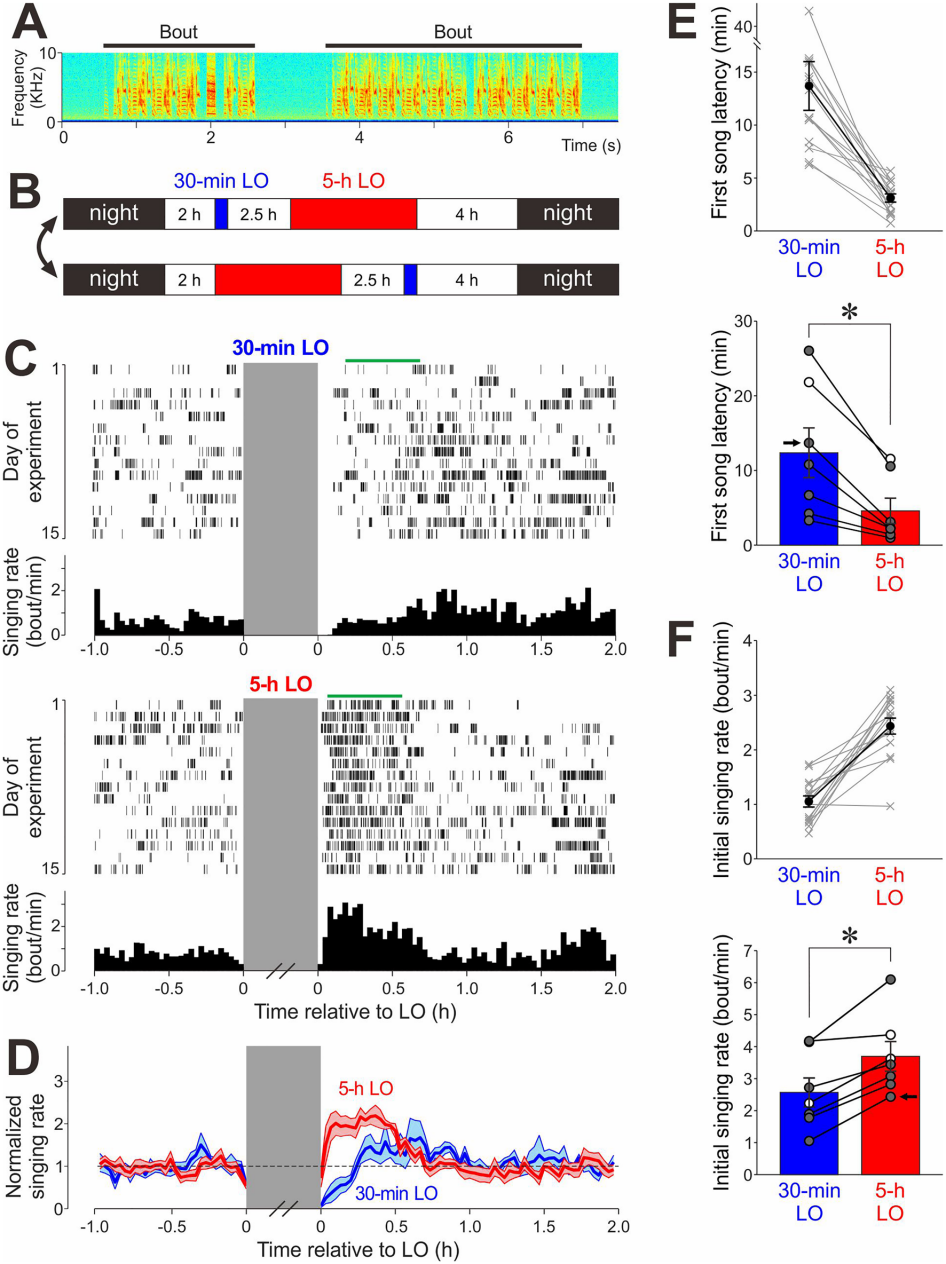
Suppression of undirected singing by turning off the ambient light increases singing motivation depending on the duration of singing suppression. (**A**) Spectrogram of undirected song in a representative bird. Horizontal bars indicate song bouts. (**B**) Daily schedule of lights-out (LO) periods. After a 2-h light period (white area) in the morning, 30-min LO (blue area) and 5-h LO (red area) were given with a 2.5-h intervening light period, followed by a 4-h light period. The order of 30-min LO and 5-h LO was switched every 1–3 days. Each row indicates the schedule on one day. (**C**) Raster plot of song bouts produced before and after 30-min LO (top) and 5-h LO (bottom) and corresponding singing rate histograms (bin size is 2 min) in a representative bird. Green horizontal lines indicate the 30-min periods in which the initial singing rates were measured for the 1st experimental day (see “Methods”). Note that the bird started singing sooner and with higher rates after 5-h LO compared to after 30-min LO. (**D**) Time course of instantaneous singing rate before and after 30-min (blue) and 5-h LO (red), normalized to the mean singing rate before LO (mean ± SEM, n = 7 birds). (**E**) The first song latencies after 5-h LO and those after 30-min LO in the bird shown in (**C**) (*top*) and in all the birds examined (n = 7 birds, *bottom*). Gray lines on *top* indicate data from single days, and the black line indicates their mean ± SEM. Circles on *bottom* indicate mean data from individual birds and bars and whiskers indicate mean + /− SEM across birds; filled circles indicate statistical significance for individual birds (*p* < 0.05). Arrow indicates the bird shown in the *top*. As a group, first song latencies were significantly shorter after 5-h LO compared to 30-min LO (*p = 0.016). (**F**) Initial singing rates after 5-h LO and those after 30-min LO in the bird shown in (**C**) (*top*) and in all the birds examined (n = 7 birds, *bottom*). Conventions are same as in (**E**). Initial singing rates were significantly greater after 5-h LO compared to 30-min LO (*p = 0.016).

The singing behavior of songbirds can provide a useful biological model for examining intrinsic motivation. Songbirds typically produce songs in the presence of a conspecific to attract mates and to repel competitors, but many birds also sing in the absence of any apparent recipients. Male zebra finches spontaneously produce hundreds of songs a day throughout their life even when they are isolated from other individuals (Fig. 1A)^5–8^. Such spontaneous song production in a solo context, referred to as “undirected singing,” is thought to partly serve as vocal practice by which birds develop and optimize a complex motor skill, song^9–12^. Unlike general motor behaviors extensively studied in other laboratory animals, undirected singing is generated in the absence of any immediate external rewards, such as food or copulation, and appears to be driven by intrinsic motivation to obtain internal rewards (a positive affective state)^13^. Undirected singing, therefore, provides a unique opportunity to study the mechanisms of intrinsic motivation that drives the learning and production of complex motor skills.

Despite extensive neuroethological studies of birdsong over the last several decades, the neural substrates underlying intrinsic motivation that drive undirected singing are still poorly understood. One major obstacle has been the difficulty associated with manipulating the intrinsic motivation to sing. Singing motivation, which is typically measured with the amount of singing in a certain period, can be artificially manipulated by hormone or drug administration^14–16^. However, it is critical to have a more natural, behavioral method to specifically manipulate the motivation to produce undirected song. Another obstacle in the study of intrinsic motivation is separating the motivation to sing from the internal reward associated with singing itself^13^. It is generally accepted that motivation, the desire to perform behavior to obtain a reward (a state of “wanting”), is closely linked to but clearly distinct from the hedonic reaction to reward itself (a state of “liking”). These two processes are also believed to be mediated by different neuromodulators in mammals, “wanting” by dopamine and “liking” by opioid peptides^4,17–20^. Previous studies in songbirds have shown that the levels of dopamine- or opioid-related signals are correlated with the amount of undirected singing^16,21–26^: more dopamine or more opioids is correlated with more undirected singing. However, given that singing itself acts as an internal reward^13^, which could increase the motivation to sing, it is unclear whether these neuromodulatory changes are involved in a process of singing motivation (“wanting”) and/or in a hedonic reaction to singing-associated reward (“liking”). A recent study has shown the involvement of opioids in the process of singing-associated reward^27^, but the neuromodulators mediating the process of singing motivation remain poorly understood. To better understand this, it is critical to develop an experimental procedure to measure singing motivation independent of the act of singing.

Here, using adult male zebra finches, we show that intrinsic motivation to produce undirected song can be reliably enhanced by turning off the ambient light, a simple behavioral and naturalistic manipulation that suppresses song production. This enhancement of singing motivation, indicated by reduced latencies to the first song after singing suppression and increased singing rates during the post-suppression period, depended on durations of singing suppression as well as on birds’ age. Moreover, by using first song latencies after singing suppression as a measure of singing motivation independent of singing-associated reward, we demonstrate that singing motivation is critically regulated by dopamine through D2 receptors.

## Results

### Long-term suppression of undirected singing increases singing motivation

In young adult male zebra finches (87–119 days post-hatch [dph]), we examined how temporary suppression of undirected singing affects singing motivation by assessing singing behavior immediately after the singing suppression period. Undirected singing was suppressed by turning off the light in the sound recording chambers, during the daytime, for a short (30 min) and a long (5 h) period (separated by a 2.5-h light period; Fig. 1B). To eliminate potential influences of circadian rhythm, the order of the 30-min and 5-h lights-out (LO) periods was switched every 1–3 days (individual birds received both 30-min and 5-h LO 7–27 times [median = 16.0]). No song production was observed during the LO periods regardless of their duration.

Interestingly, when we compared singing immediately after the offset of 30-min LO and 5-h LO periods, we found that birds sang much sooner and more intensely after 5-h LO (Fig. 1C–F, Supplementary Fig. 1). On average, instantaneous singing rates (calculated using 2-min time bins) rapidly increased, to almost double baseline singing rates, only after 5-h LO, and then gradually returned to baseline levels ~ 1 h after LO offset (Fig. 1C–D). Also, the latency to the first song after end of LO, which we refer to as “first song latency,” was significantly shorter after 5-h LO compared to 30-min LO in 6 out of 7 birds (Fig. 1E; *p* < 0.05 for each bird; *p* = 0.016 for group data [n = 7 birds, W = 28], Wilcoxon signed-rank test). Moreover, the mean singing rates measured over a 30-min period starting at the first song following each LO period (green bars in Fig. 1C), which we refer to as “initial singing rates,” were significantly higher after the 5-h LO than after the 30-min LO in 5 out of 7 birds (Fig. 1F; *p* = 0.016 for group data; W = 0). Because birds with higher singing motivation should show shorter first song latency and higher initial singing rate, these results suggest that intrinsic motivation for undirected singing increases when singing is suppressed for a relatively long time. Behavioral latencies and frequencies have generally been used to quantify levels of motivation to initiate the behavior in other non-human animals as well^19,28–30^.

### Singing motivation monotonically increases depending on the duration of singing suppression

**Figure 2:**
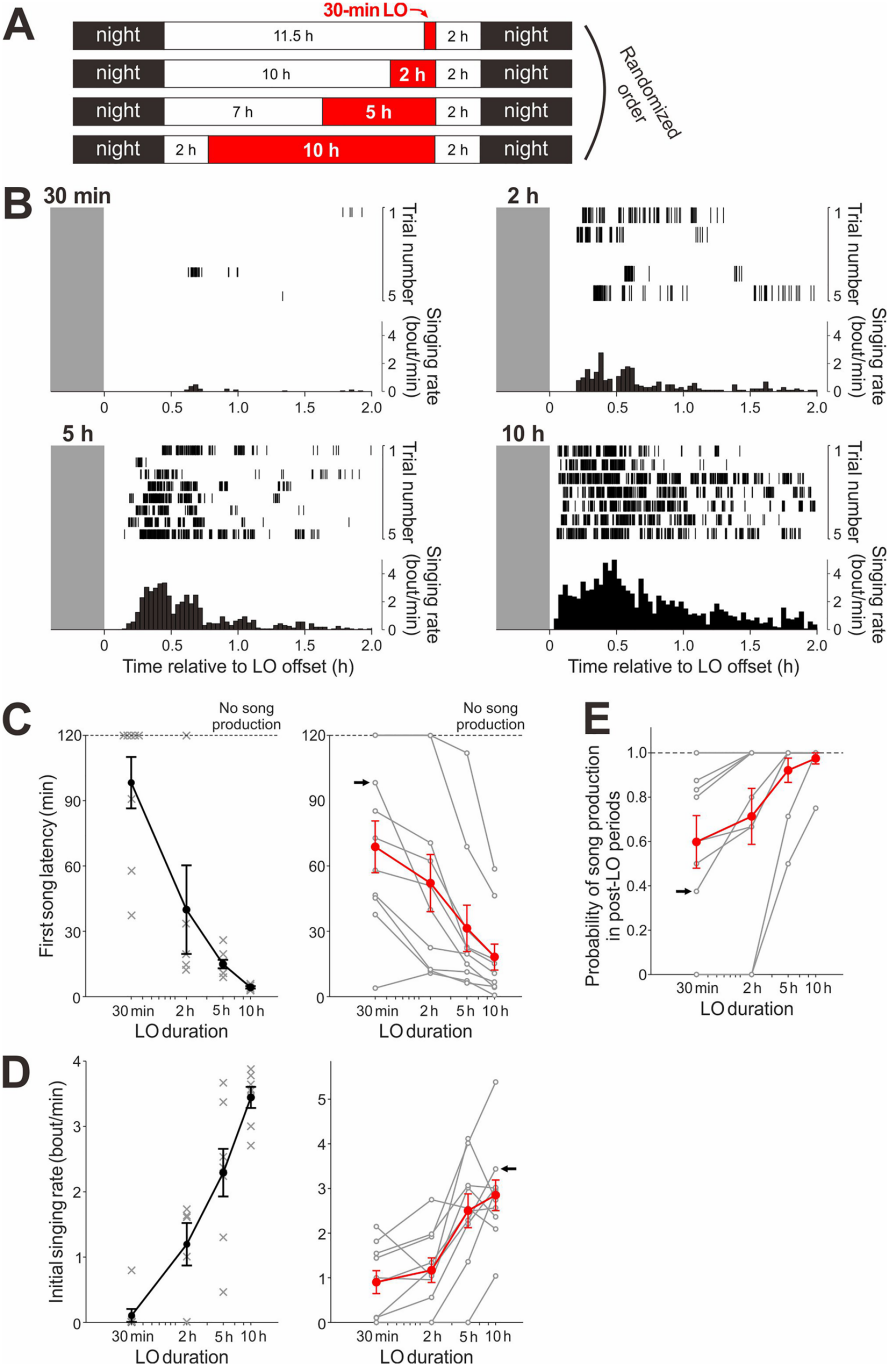
Singing motivation monotonically increases depending on the duration of singing suppression. (**A**) Daily schedule of LO periods (red areas) with four different durations (30 min, 2 h, 5 h, and 10 h). On each day, a single LO period with one of the four different durations was given with the offset at 2 h before the nighttime; the onset time was varied depending on the LO duration. Birds received LO periods with four different durations in a randomized order. (**B**) Raster plots of song bouts produced after LO periods with variable durations (top) and corresponding singing rate histograms (bottom) in a representative bird. (**C**) First song latencies plotted against LO durations from the bird shown in (**B**) (*left*) and from all the birds examined (*right;* n = 10 birds). In the left panel, gray crosses represent data from a single day and black lines represent mean + /− SEM across days; if no song production was observed over the 2-h time window after LO, the data was assigned a time of 120 min (dashed line). In the right panel, gray lines indicate mean data for individual birds and red lines represent mean + /− SEM across all birds; arrow indicates the bird shown in the *left*. There was a significant trend of monotonic decreases in first song latency as LO duration increased (*p* = 0.010, one-way ANOVA). (**D**) Initial singing rates plotted against LO duration from the bird shown in (**B**) (*left*) and from all the birds examined (*right*; n = 10 birds). Conventions are same as in (**C**). Initial singing rates monotonically increased as LO duration increased (*p* = 0.0001). (**E**) Probability of song production (the number of post-LO periods during which a bird sung divided by the total number of post-LO periods examined for each bird) plotted against different LO durations. Conventions are same as in (**C**)* right*. There was a significant trend of monotonic increases in song production probability as LO duration increased (*p* = 0.020).

We further characterized increases in singing motivation caused by singing suppression. Birds were subjected to LO periods with four different durations (30 min, 2 h, 5 h, and 10 h) in random order (4–8 times for each duration [median = 6.0]). On each experimental day, birds received a single LO period, with one of the four durations. Independent of the duration, LO periods always ended at 2 h before nighttime to exclude possible circadian effects (Fig. 2A). We observed a clear trend that birds sang sooner at higher rates after longer LO durations: first song latencies monotonically decreased as LO duration increased (Fig. 2B, C; n = 10 birds; *p* = 0.010, *F*(3,36) = 4.29, one-way ANOVA; when birds did not produce any songs during the 2-h post-LO period, the data of first song latency was assigned a time of 2 h [120 min]; see Methods); likewise, initial singing rates monotonically increased as LO durations increased (Fig. 2B, D; *p* = 0.0001, *F*(3,36) = 9.1; when birds did not produce any songs, the data of the initial singing rate was assigned zero). Moreover, the probability of singing during the post-LO periods also monotonically increased with increasing LO duration (Fig. 2B, E; *p* = 0.020, *F*(3,36) = 3.7): while birds often produced no songs after short LO periods (30-min and 2-h LO), most birds reliably sang after 10-h LO periods on all experiment days (Fig. 2B, E). These results demonstrate that intrinsic motivation for undirected singing can be easily manipulated over a wide range simply by changing the duration of LO periods, providing a useful tool for studying the neural mechanisms of singing motivation in songbirds.

### Singing motivation can be enhanced by singing suppression even under light conditions

**Figure 3:**
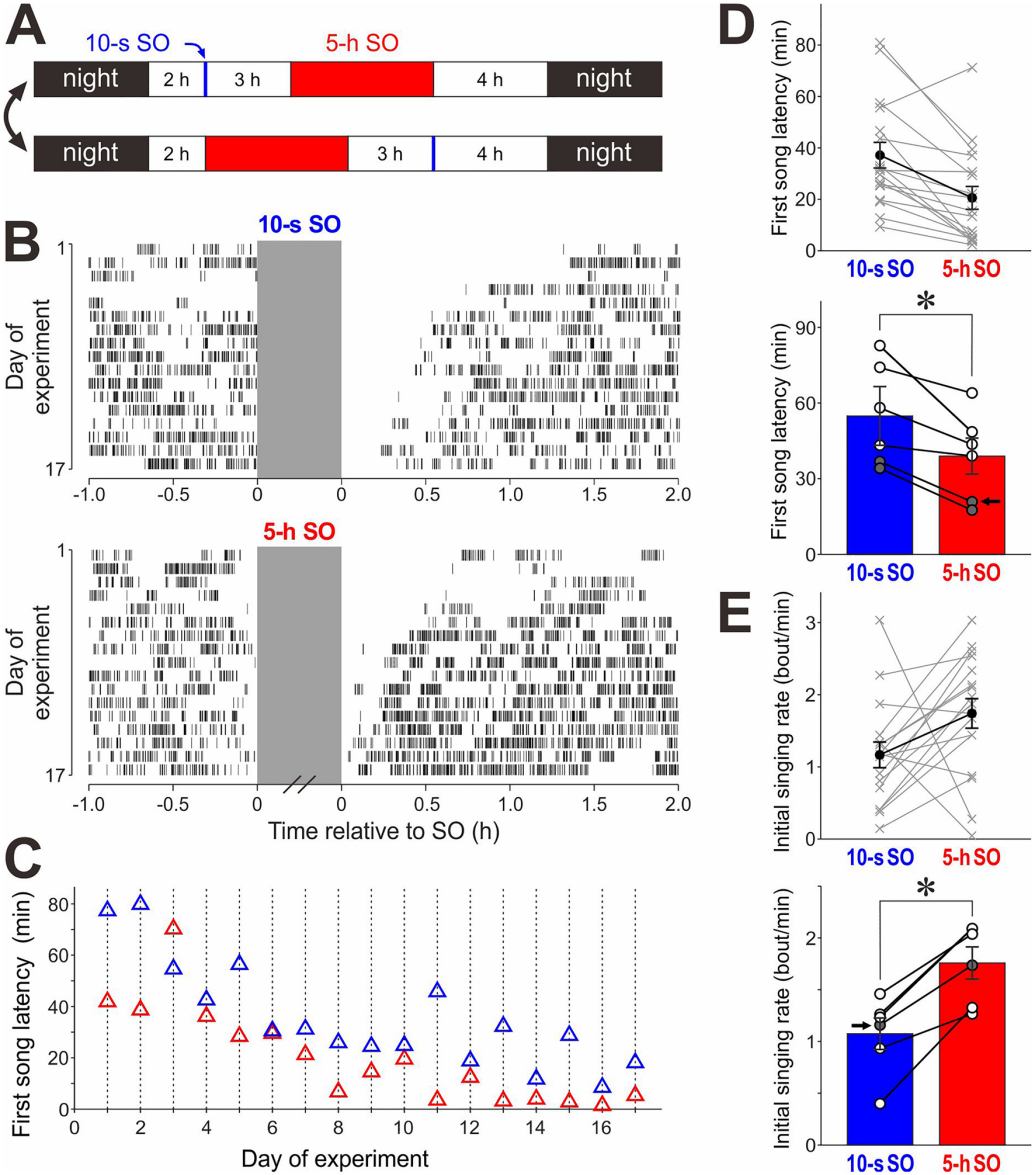
Suppression of undirected singing under light conditions increases singing motivation in a manner similar to singing suppression by lights-out. (**A**) Daily schedule of singing suppression. At 2 h after turning on the light in the morning, brief (~ 10-s, blue) and 5-h (red) singing obstructions (SOs) were given with a 3-h interval. The order of 10-s SO and 5-h SO was switched every 1–2 days. (**B**) Raster plot of song bouts produced before and after 10-s (top) and 5-h (bottom) SOs in a representative bird. Conventions are same as in Fig. 1C. (**C**) First song latencies after 10-s SOs (blue) and 5-h SOs (red) on individual experiment days, obtained from the data shown in (**B**). (**D**) First song latencies after 10-s SOs and those after 5-h SOs in the bird shown in (**B**, **C**) (*top*) and in all the birds examined (n = 6 birds, *bottom*). Conventions are same as in Fig. 1E. As a group, first song latencies were significantly shorter after 5-h SO compared to 10-s SO (*p = 0.03). (**E**) Initial singing rates after 5-h SOs and those after 10-s SOs in the bird shown in (**B**, **C**) (*top*) and in all the birds examined (n = 6 birds, *bottom*). Initial singing rates were significantly greater after 5-h SO compared to 10-s SO (*p = 0.03).

The amount of singing in most songbirds is also regulated by circadian rhythms. Birds often sing a lot early in the morning after a long dark period^31^. Given that we suppressed singing by turning off the lights, we cannot exclude the possibility that a prolonged dark condition, and not song suppression, influences singing motivation and subsequent singing. To test this possibility, we suppressed singing under light conditions by physically interfering with singing posture using a method previously reported^32^ (see “Methods”). Singing was suppressed for short (10-s) and long (5-h) time periods with a schedule similar to that in the LO experiments described in Fig. 1B (individual birds received both 10-s and 5-h singing obstruction (SO) 16–18 times [median = 17.5]; Fig. 3A). We observed gradual decreases in first song latencies over days after both 10-s and 5-h SO periods (Fig. 3B), presumably reflecting habituation of the birds to the SO procedure. Nevertheless, there was a strong trend of enhanced singing motivation after long suppression compared to short suppression just like the LO experiments: for individual experimental days, first song latencies tended to be shorter after 5-h SO than after 10-s SO (Fig. 3C, D top). As a group, first song latencies were significantly shorter after 5-h SO than after 10-s SO (Fig. 3D bottom; n = 6 birds; *p* = 0.03, W = 21, Wilcoxon signed-rank test). Likewise, initial singing rates were significantly greater after 5-h SO than after 10-s SO (Fig. 3E; n = 6 birds; *p* = 0.03, W = 0). These results indicate that singing motivation can be enhanced by singing suppression even under light conditions, thus confirming that singing suppression, and not dark conditions per se, is critical for enhancing the intrinsic motivation for undirected singing.

### Suppression-dependent enhancement of singing motivation depends on age

**Figure 4:**
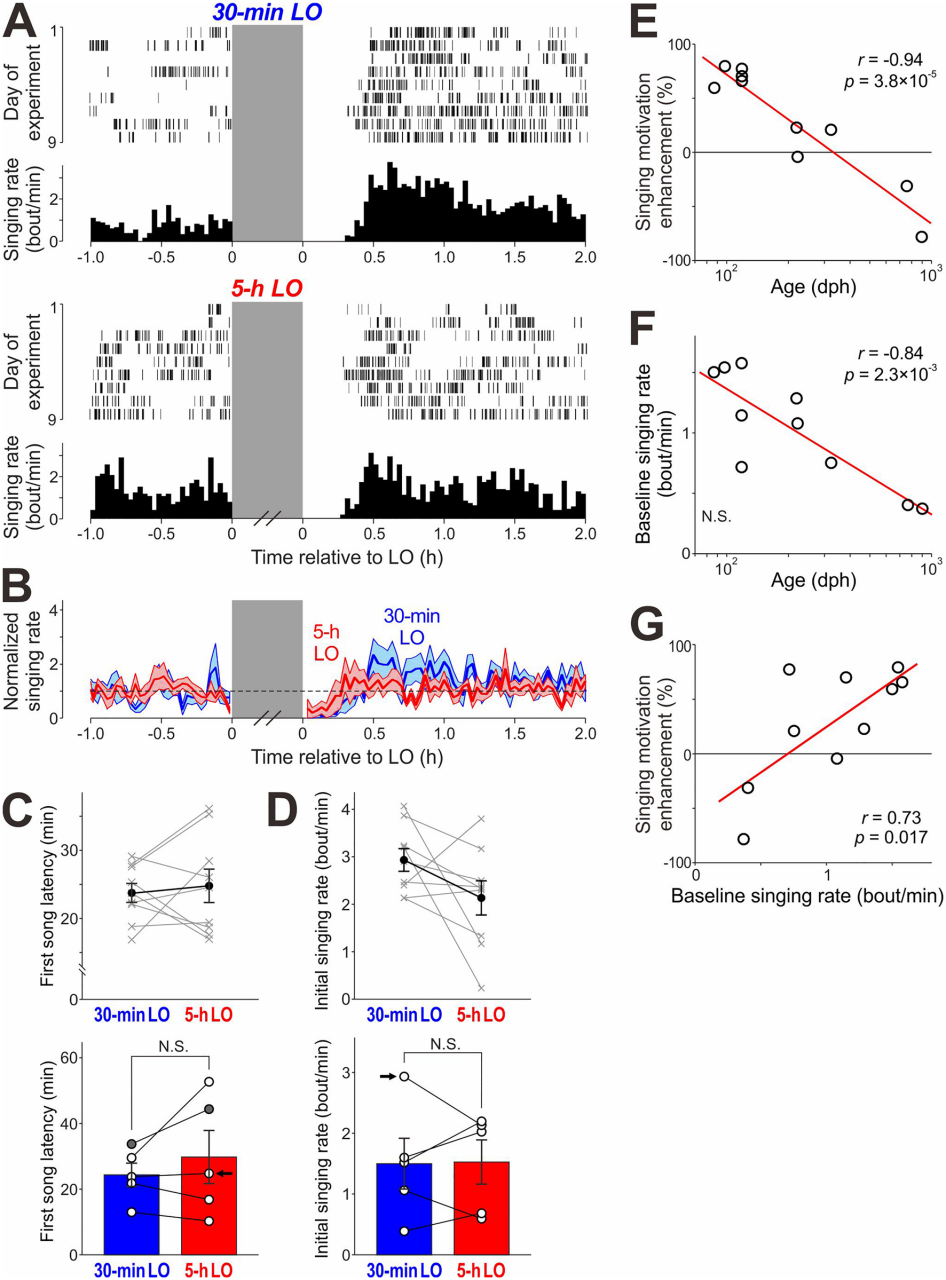
Singing suppression-induced enhancement of singing motivation depends on age. (**A**) Raster plot of song bouts produced before and after 30-min LO (top) and 5-h LO (bottom) and corresponding singing rate histograms in a relatively old adult bird (222 dph on the first day of the experiment). (**B**) Time course of instantaneous singing rate before and after 30-min (blue) and 5-h LO (red), normalized to the mean singing rate before LO (mean ± SEM, n = 5 birds). (**C**) The first song latencies after 5-h LO and those after 30-min LO in the bird shown in (**A**) (*top*) and in all the birds examined (n = 5 birds, *bottom*). Conventions are same as in Fig. 1E. As a group, first song latencies were not significantly different between 5-h LO and 30-min LO (*p* = 0.63). (**D**) Initial singing rates after 5-h LO and those after 30-min LO in the bird shown in (**A**) (*top*) and in all the birds examined (n = 5 birds, *bottom*). There was no significant difference between 5-h LO and 30-min LO (*p* = 1). (**E**) Magnitude of singing motivation enhancement (see “Methods”) plotted against birds’ age (n = 10 birds including both young and old adults). The red line indicates linear regression. (**F**) Baseline singing rates plotted against birds’ age. (**G**) Magnitude of singing motivation enhancement plotted against baseline singing rates.

All the experiments described so far were done with relatively young adult zebra finches (87–119 dph). To examine age dependence of the suppression-induced enhancement of singing motivation, we suppressed singing in older birds (219–897 dph) using the LO procedure described in Fig. 1B (individual birds received both 30-min and 5-h LO 9–14 times [median = 9.0]). Surprisingly, we observed no difference in singing behavior between 30-min LO and 5-h LO (Fig. 4A–D). In stark contrast to the striking and consistent decreases in first song latencies after 5-h LO compared to 30-min LO in young adult birds (Fig. 1C–E), no significant difference was observed in 4 out of 5 older adult birds examined (*p* > 0.05 for each bird, Wilcoxon signed-rank test; Fig. 4A–C); the other bird showed even longer latencies after 5-h LO compared to those after 30-min LO (*p* = 0.020); as a group, no significant difference was observed between 30-min LO and 5-h LO (Fig. 4C *bottom*, n = 5, *p* = 0.63, W = 5, Wilcoxon signed-rank test). Similarly, initial singing rates after LO were not significantly different between 30-min LO and 5-h LO in 5 out of 5 older birds or as a group (Fig. 4D; *p* = 1, W = 8 for the group data). In fact, the extent to which singing motivation was enhanced after 5-h LO as compared to 30-min LO, as measured by relative change in mean first song latencies (see “Methods”), was strongly correlated with age (Fig. 4E; n = 10 birds, *r* = 0.94, *p* = 3.8 × 10^–5^). These results show that the enhancement of singing motivation after song suppression was strongly dependent on the age of the bird: younger the bird, greater the enhancement and older the bird, lesser the enhancement.

Previous studies have shown that the daily amount of undirected song is relatively high in young birds and gradually decreases with age, even from young adults to old adults^7,8^, suggesting an age-dependent decline in overall motivation for undirected singing. We hypothesized that such an age-dependent decline of overall singing motivation contributed to the observed age dependence of singing motivation enhancement after long-term singing suppression. To test this hypothesis, we measured baseline singing rate (mean singing rates over 1-h periods immediately before LO periods) in both young and old adult birds as a measure of baseline singing motivation and compared it with age and with the magnitude of singing motivation enhancement. In accord with a previous study showing an age-dependent decrease in daily singing amounts with age ranges similar to those of our birds^7^, baseline singing rates immediately before LO periods gradually decreased with age (Fig. 4F, n = 10 birds, *r* = − 0.84, *p* = 2.3 × 10^–3^). We found that these baseline singing rates are positively correlated with the magnitude of singing motivation enhancement caused by singing suppression (Fig. 4G, n = 10 birds, *r* = 0.73, *p* = 0.017): younger adult birds exhibited higher baseline singing rates and greater increases in singing motivation after singing suppression compared to older birds. These results are consistent with the hypothesis that the age-dependent decline of overall singing motivation contributes to the age dependence of singing motivation enhancement caused by long-term singing suppression.

### Effects of dopamine and opioid receptor antagonists on intrinsic motivation for undirected singing, quantified as first song latencies

Previous studies have demonstrated the regulation of undirected singing by at least two neuromodulators, dopamine and opioids. Singing rates were significantly correlated with levels of dopamine- or opioid-related signals^16,21–26^. Although these correlative results suggest the involvement of dopamine and opioids in undirected singing, whether those neuromodulators critically contribute to the motivation to sing (a state of “wanting”) and/or to intrinsic reward associated with the act of singing (a state of “liking”) remains unclear, as singing rates analyzed in previous studies are closely linked to both motivation and reward processes. For example, an increase in singing rate could reflect an increased level of singing motivation and/or an increase in singing-associate reward. It is difficult to tell these two apart by just measuring singing rates. However, our measure of first song latency is more likely to reflect singing motivation, as it is unlikely to be influenced directly by any processes following the act of singing, such as singing-associated reward, especially when singing is suppressed for a relatively long time. Taking advantage of this measure, we assessed the direct contributions of dopamine and opioids to undirected singing motivation (independent of singing-associated reward) by administering antagonists of these neuromodulators.

**Figure 5:**
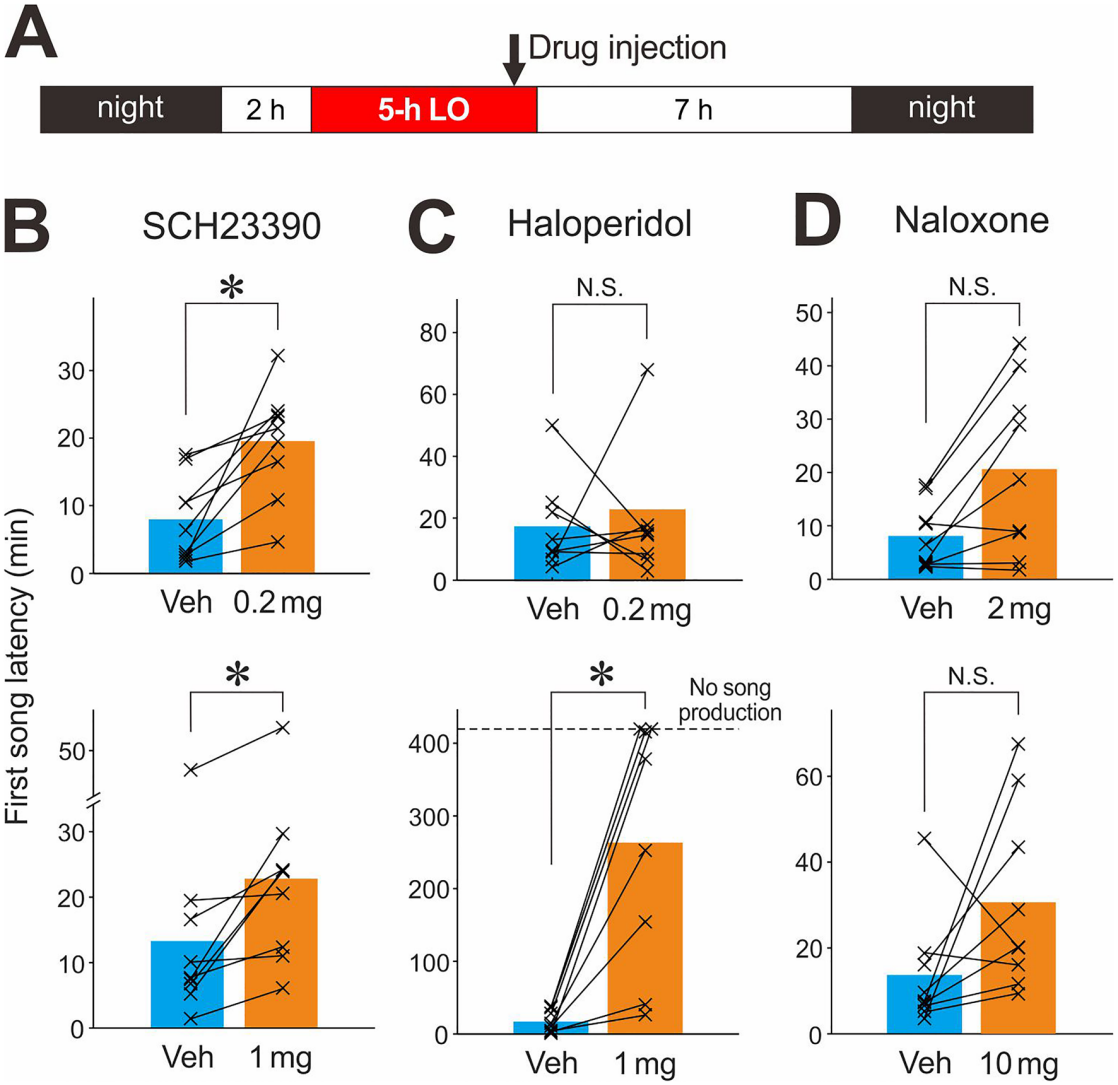
Effects of systemic injections of dopamine or opioid antagonists on first song latencies after 5-h LO in young adult birds. (**A**) Schedules of drug injections. Drugs or their vehicles were injected at 30-min preceding the offset of 5-h LO periods. (**B**) Comparisons of first song latencies between a dopamine D1 receptor antagonist SCH23390 with lower (0.2 mg/kg, *top*) and higher (1 mg/kg, *bottom*) doses and its vehicle (‘Veh’). Each line indicates a single bird. Injections of both lower and higher doses significantly increased first song latencies compared to the vehicle (*p = 0.004, significance level α was corrected to 0.0083 with a Holm-Bonferroni correction for multiple comparisons). (**C**) Comparisons between a dopamine D2 receptor antagonist haloperidol with lower (0.2 mg/kg, *top*) and higher (1 mg/kg, *bottom*) doses and its vehicle. Injections of the higher dose, but not the lower dose, greatly prolonged the first song latencies (*p = 0.008, corrected α = 0.0125). If no song production was observed during the post-LO period (7-h duration), the data was assigned a time of 7 h (420 min, dashed line). (**D**) Comparisons between an opioid antagonist naloxone with lower (2 mg/kg, *top*) and higher (10 mg/kg, *bottom*) doses and its vehicle. The effects of naloxone were not significant for either dose (*p* = 0.04 and corrected α = 0.0167 for lower dose; *p* = 0.07 and corrected α = 0.025 for higher dose).

We systemically injected antagonists of dopamine or opioid receptors at the end of 5-h LO periods (30 min before the offset of LO periods) and assessed their effects on first song latencies during the subsequent light period (7-h duration; Fig. 5A). Doses of the drugs were based on previous studies using the same drugs in songbirds and chickens^16,33–35^. Compared with vehicle injections, we found that low (0.2 mg/kg) and high (1 mg/kg) doses of a dopamine D1 receptor antagonist, SCH23390, moderately but significantly increased the first song latencies after 5-h LO (Fig. 5B; n = 9 birds, *p* = 0.004, W = 0 for both low and high doses, Wilcoxon signed-rank test with a Holm-Bonferroni correction for multiple comparisons, corrected significance level α = 0.0083; effect size [Hedges' g] = 1.69 for low dose and 0.67 for high dose). In contrast with SCH23390 injections, injections of a dopamine D2 receptor antagonist, haloperidol, dramatically increased the first song latencies in a dose-dependent manner; first song latencies were much longer with a higher dose (1 mg/kg) but not lower dose (0.2 mg/kg) when compared with vehicle injections (Fig. 5C; n = 8 birds, *p* = 0.95, α = 0.05, W = 27, effect size = 0.11 for lower dose; n = 9, *p* = 0.008, corrected α = 0.0125, W = 0, effect size = 2.16 for higher dose). In addition, 3 out of 9 birds with the higher dose haloperidol injections did not produce any songs during the post-LO periods (7-h duration), whereas all birds with vehicle injections produced songs during the same period (dashed line in Fig. 5C *bottom*). These results suggest that dopamine plays a critical role in regulating intrinsic motivation for undirected singing through D2 receptors. Although D1 receptors also appear to be involved in undirected singing motivation, given the relatively small effect of SCH23390 and its potential influence on D2 receptor signaling^36^, further studies are needed to determine the contribution of D1 receptors (see also Discussion). We also tested the possibility that these dopamine receptor antagonists affect general motor behavior by examining the number of hopping and flying over a 30-min period following 5-h LO, but found no significant effects for either drug at any dose (Supplemental Fig. 2). Thus, the increasing effects of these drugs on first song latencies are not simply due to decreases in general motor behavior.

In contrast to the dopamine D2 receptor antagonist haloperidol, the general opioid receptor antagonist naloxone did not have strong effects on first song latencies. Although there appear to be a trend of longer first song latencies with naloxone injections (both the low [2 mg/kg] and high [10 mg/kg] doses) compared to those with vehicle injections (effect size = 1.03 and 0.99 for low and high doses, respectively), the differences were not statistically significant (Fig. 5D; n = 9 birds, *p* = 0.04, corrected α = 0.0167 and W = 5 for lower dose; *p* = 0.07, corrected α = 0.025 and W = 7 for higher dose). Naloxone did not significantly affect non-vocal behaviors over a 30-min period following 5-h LO either (Supplementary Fig. 2), as reported in a previous study that used similar doses and experimental paradigms^16^.

### Effects of dopamine and opioid receptor antagonists on initial singing rates

**Figure 6:**
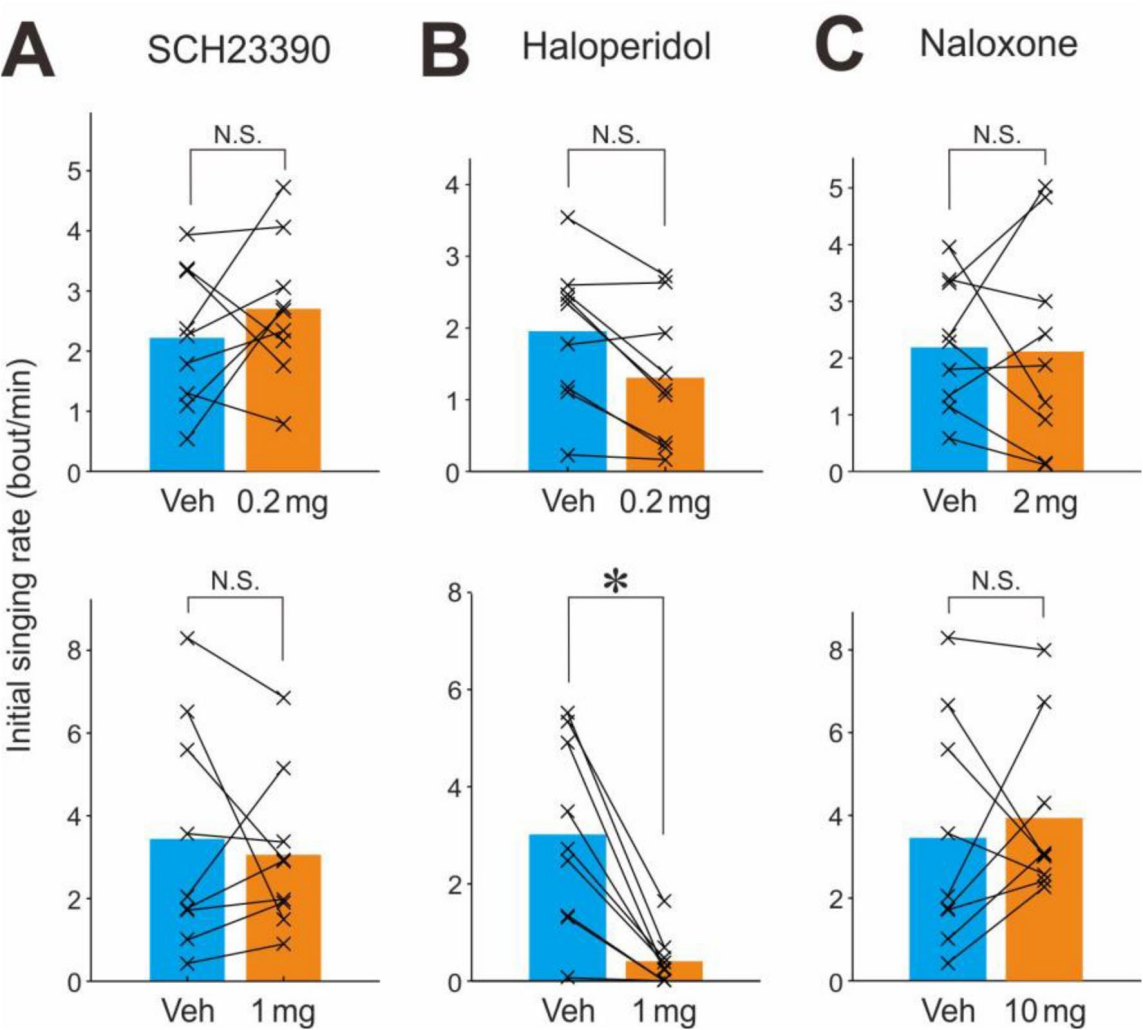
Effects of systemic injections of dopamine or opioid antagonists on initial singing rates after 5-h LO in young adult birds. Conventions are same as in Fig. 5. Only the higher dose (1 mg/kg) of haloperidol significantly decreased initial singing rates (*p = 0.004 and corrected α = 0.008).

We also examined the effects of dopamine and opioid receptor antagonists on the initial singing rate, which, unlike the first song latency, is likely to reflect not only intrinsic singing motivation but also singing-associated reward. We found that neither low (0.2 mg/kg) nor high (1 mg/kg) dose of SCH23390 had significant effects on initial singing rates (Fig. 6A; n = 9 birds, *p* = 0.3, corrected α = 0.0125 and W = 13.5 for lower dose; n = 9 birds, *p* = 1.0, corrected α = 0.05 and W = 23 for higher dose; Wilcoxon signed-rank test with a Holm-Bonferroni correction for multiple comparisons), sharply contrasting with their increasing effects on the first song latency (Fig. 5B). Because singing rates directly reflect the degree of singing motivation (and probably indirectly reflect singing-associated reward), these results suggest that singing motivation that has been suppressed by this drug mostly recovers by the time the birds produce the first song following the LO period. Given that the drug and the vehicle were injected 30 min prior to the offset of the LO period and that many birds resumed singing ~ 20 min after the LO offset (see Fig. 5A and B), it is likely that the drug effect to suppress singing motivation mostly wears off during this period. In contrast, the high dose (0.2 mg/kg), but not the low dose (1 mg/kg), of haloperidol significantly decreased the initial singing rate (Fig. 6B; n = 9 birds, *p* = 0.03, corrected α = 0.01 and W = 41 for lower dose; n = 9 birds, *p* = 0.004, corrected α = 0.008 and W = 45 for higher dose; if no songs were produced during the 7-h, post-LO periods, the data of the initial singing rate was assigned zero). This inhibitory effect of the high dose of haloperidol on initial singing rate is not surprising given its long-lasting inhibitory effect on singing motivation reflected by markedly-prolonged first song latencies (Fig. 5C): it is likely that singing motivation is still partly suppressed even after the birds resumed singing following LO periods, resulting in reduced initial singing rates. We also found that neither the low (2 mg/kg) nor high (10 mg/kg) dose of naloxone had significant effects on initial singing rates (Fig. 6C; n = 9 birds, *p* = 0.9, corrected α = 0.025 and W = 24 for lower dose; n = 9 birds, *p* = 0.7, corrected α = 0.017 and W = 19 for higher dose). Although these results appear to be inconsistent with a previous study showing dose-dependent inhibitory effects of similar doses of naloxone on undirected singing^16^, this discrepancy can also be explained by the time gap between the drug injection and the measurement of singing rates in our experiments as explained for our SCH23390 experiments above. This interpretation is supported by the fact that naloxone crosses the blood brain barrier and exits the brain rapidly^37^.

## Discussion

In the present study, we highlight the advantage of undirected singing in zebra finches as a model system to study intrinsic motivation for a complex, learned behavior. We found that temporary suppression of spontaneous undirected singing, either by turning off the ambient light or by physically obstructing singing, dramatically increased intrinsic motivation for singing as quantified by singing latencies and frequencies. This increase in motivation depended on the duration of singing suppression: longer the suppression of singing, sooner and more intense was the singing after release from suppression. We also found that suppression-induced enhancement of singing motivation depended on age, presumably due to an age-dependent decline of baseline singing motivation. Finally, we revealed that singing motivation is critically regulated by dopamine through D2 receptors. Taken together, our findings provide a simple and useful experimental tool to manipulate and measure singing motivation independent of singing-associated reward and offer novel insights into the mechanisms underlying intrinsic motivation for vocal practice in songbirds.

Spontaneous and intense singing of songbirds in the absence of apparent recipients, such as undirected singing of zebra finches, has long been of immense interest to biologists as the function of undirected singing remains unclear. Accumulating evidence in zebra finches and Bengalese finches indicates that undirected singing serves, at least in part, as vocal practice by which birds develop and optimize song structures^12,38–41^. Because such vocal practice would ultimately increase the effectiveness of song during future interaction with a mate, maintaining a high level of motivation for undirected singing to routinely optimize song structure appears to be critical for reproduction success. Our findings of enhanced singing motivation after long periods of singing suppression may be explained by this model: immediate and intense singing following long periods of singing suppression may compensate for the loss of vocal practice during singing suppression and enable birds to quickly and thoroughly re-evaluate and re-optimize song structure to prepare for future courtship activity. Future research should examine whether daily undirected singing is critically required for the maintenance of song structure by suppressing undirected singing for multiple days and assessing its effect on the detailed structure of song. Additionally, similar mechanisms could account for the observed age-dependent declines of overall singing rate and of singing motivation enhancement caused by singing suppression. Although young adult zebra finches maintain and optimize song structure through vocal practice, song plasticity gradually declines with age^10,11,42^. Given the reduced plasticity of song structure in older adult birds, it is possible that less vocal practice is sufficient to maintain song structure even after relatively long singing suppression. Consistent with this line of thinking, birds may decrease undirected singing motivation and daily song amount with age by constantly evaluating how much vocal practice is required to maintain song structure and by adaptively adjusting singing motivation in order to decrease energy consumption and/or the risk of predation caused by undirected singing. Alternatively or in addition, because the act of undirected singing is rewarding^13^, lower singing rates resulting from lower singing motivation in old birds may simply be caused by age-dependent decline of intrinsic reward associated with undirected singing which could be genetically encoded in the brain.

Enhancement of singing motivation depending on duration of singing suppression is reminiscent of the so-called “Lorentz’ psychohydraulic model”^43^, a classic model of animal motivation. In this model, a fluid representing action-specific energy (i.e. motivational drive) builds up in a reservoir over time if the behavior is not being executed, leading to the eventual opening of a valve of the reservoir depending on external factors that modulate the valve opening; the flow of action-specific energy out of the reservoir into a bucket underneath represents the execution of the behavior with fixed action patterns. Although this model is over-simplified in comparison with the modern view of motivation, suppression-induced enhancement of undirected singing motivation that we observed nicely fits the main concept of this model: the fluid in the reservoir represents motivation for undirected singing and accumulation of the fluid by long-term suppression of singing results in immediate and robust expression of singing behavior when the suppression was released; the external factor represents ambient light in our LO experiments. Thus, our results underscore the importance of undirected singing as a tractable model system for studying the fundamental mechanisms of animal motivation.

Our results of pharmacological manipulation of dopamine or opioid signaling provide a significant advance in our understanding of the mechanisms underlying the spontaneous production of undirected song. Previous studies have demonstrated that both dopamine and opioids are associated with the production of undirected song^16,21–26,33^, and opioids have recently been demonstrated to contribute to the process of singing-associated regard (a state of “liking”)^27^. However, it was unclear whether dopamine contributes to the motivation to sing (a state of “wanting”) and/or singing-associated reward. Using a measure “first song latency,” which quantifies the degree of singing motivation independent of singing-associated reward, we revealed that dopamine contributes to the regulation of singing motivation through D2 receptors. Although our results of naloxone treatment are not clear and do not allow for a strong conclusion regarding the contribution of opioids to singing motivation, the critical contribution of opioids to singing-associated reward shown by a recent study^27^ and the contribution of dopamine to singing motivation shown by our current results highlight striking parallels with the mechanisms of reward-associated behaviors in mammals. A large body of research in mammals indicates that dopamine primarily underlies anticipatory, motivated state whereas opioids underlies hedonic pleasure or reward (for review,^4,18–20^).

Future studies should identify which neural circuits are modulated by dopamine to regulate singing motivation. Previous studies demonstrated that dopamine-related signals are correlated with the production of undirected singing in several brain areas including Area X, the medial preoptic area (mPOA), the ventral tegmental area (VTA), and septum^21,22,26,44^, but it remains unclear whether those areas are involved in singing motivation. Although dopaminergic inputs from VTA to a song-specialized basal ganglia nucleus Area X are activated during undirected singing^21,44^, they are unlikely to underlie singing motivation based on the following findings: (1) lesions of the VTA-Area X projections or of Area X itself do not abolish undirected singing^45–50^; (2) neither stimulation nor inhibition of VTA-Area X axon terminals alters undirected singing rate^51^; and (3) the VTA-Area X projections fire during, but not before, singing to encode performance error regarding ongoing song quality^51–53^, which is similar to reward prediction error in mammalian VTA dopamine neurons observed during reward-seeking tasks^54^. Independent of reward-dependent firing of VTA dopaminergic neurons, gradual increases in extracellular dopamine levels have recently been observed in mammalian striatum when animals approach a reward site^30,55^, suggesting a critical role for slow time scale dopamine releases in motivation for reward-seeking behavior. Similar mechanisms might be involved in regulating intrinsic motivation for undirected singing in songbirds.

Relative contributions of different subtypes of dopamine receptors to undirected singing motivation is also needed to be determined. Although our systemic injections of a D1 receptor antagonist SCH23390 significantly increased the first song latencies (Fig. 5B), given that SCH23390 can also block GIRK channels^56^, which are downstream targets of dopamine D2 receptor signaling^36^, there is a possibility that the increases in the first song latencies following SCH23390 injections are an off-target effect and not directly related to D1 receptor signaling. Testing the effects of other types of D1 receptor antagonist such as SCH39166 as well as D1 receptor agonists would be needed to tease out the relative contributions of different dopamine receptors to singing motivation.

Intrinsic motivation has long been a major topic of interest in human psychology, but intrinsically motivated behavior was first acknowledged in the study of animal behavior^4,57^. Since then, a variety of behaviors have been investigated across a range of species, including social play behavior in rodents^58,59^. Nevertheless, neural mechanisms underlying intrinsic motivation are still largely unclear, in part due to variability and complexity of behaviors that prevent us from examining a direct link between behavior and underlying neural activity. Undirected singing in zebra finches, investigated in the present study, is a complex but highly stereotyped and quantifiable motor skill that is learned and maintained by relatively simple neural circuits specialized for singing. Given the tractable nature of zebra finch song, as well as accumulating knowledge about neural mechanisms of song production and learning, undirected singing in zebra finches provides an excellent model system to study neural circuit mechanisms of intrinsic motivation. Thus, our simple procedure to manipulate intrinsic motivation for undirected singing and the new insights into the neuromodulatory system for regulating singing motivation provide an important first step toward understanding more detailed neural mechanisms underlying intrinsic motivation for complex, learned motor behaviors.

## Methods

This study was carried out in compliance with the ARRIVE guidelines (http://www.nc3rs.org.uk/page.asp?id=1357).

### Subjects

All subjects were adult male zebra finches (*Taeniopygia guttata*, 87–897 dph). Birds were raised in our colony with their parents and siblings until ~ 60 dph and then housed with their siblings and/or other males conspecifics until the experiments started. Care and treatment of animals was reviewed and approved by the Institutional Animal Care and Use Committee (IACUC) at the Korea Brain Research Institute. All experiments were performed in accordance with relevant guidelines and regulations.

### Song recording

For song recording, birds were housed individually in sound-attenuating chambers (MC-050, Muromachi Kikai) on a 14:10-h light:dark cycle. Songs were recorded using a microphone (PRO35, Audio-Technica) positioned above the cage and a custom-written song recording program (R.O. Tachibana). Output from the microphone was amplified by a mixer (402-VLZ4, Mackie) and digitized via an audio interface (Octa-Capture UA-1010, Roland) at 44.1-kHz (16-bit). Recorded data were down-sampled to a sampling rate of 32-kHz. Recording was triggered if the program detected five consecutive sound notes, each of which was defined based on sound magnitude, duration, and intervening gap duration. Recording ended if a silent period lasted longer than 0.5 s (i.e. each song file contains a single “song bout” that is separated from other bouts by > 0.5-s silent periods). Songs were recorded throughout the day, and all song recordings were of undirected song (i.e. no female was present). Birds with sufficient singing rates (> 300 song bouts per day) were used for our experiments.

### Singing suppression

Singing was suppressed by turning off the light in the sound-attenuating chambers using digital timers in most experiments. The duration and schedule of LO periods varied depending on experimental paradigms (from 30 min to 10 h; see Results). In a subset of birds, we suppressed singing by attaching a detachable weight (17–20 g) on the birds’ necks^32^. This procedure suppresses singing by preventing birds from taking the singing posture without affecting their daily behaviors. The weight was usually supported by the floor and not carried by the bird’s neck and therefore birds continue all their daily behaviors, such as drinking, eating, grooming, and calling. The weights were attached either for 5 h or only transiently (for ~ 10 s). No birds produced any song motifs during either LO periods or periods with the weight attached.

### Song analysis

The first song latency was measured as the time interval from the offset of a singing-suppression period to the onset of the first song recorded. We visually inspected spectrograms of the sound files recorded after the singing-suppression periods to find the first file that included at least one song motif. To quantify singing motivation enhancement using this measure, first song latencies across all LO periods with the same durations (30 min or 5 h) were averaged and percent differences between them were calculated as follows:

$${\text{Singing}}\;{\text{motivation}}\;{\text{enhancement }} = { 1}00 \, * \, \left( {{\text{Latency}}_{{{3}0 \text{-} {\text{min}}}} - {\text{Latency}}_{{{5} \text{-} {\text{hr}}}} } \right) /{\text{Latency}}_{{{3}0 \text{-} {\text{min}}}} ,$$

where Latency_30-min_ and Latency_5-hr_ are mean first song latencies after 30-min LO and after 5-h, respectively. This measure will be positive when Latency_5-hr_ is shorter than Latency_30-min_, and vice versa.

To measure singing rates during the periods before and after LO, we screened all sound files recorded during those periods to exclude non-song files using a semi-automated method. Song motifs of adult zebra finches have highly stereotyped temporal structure, which is clearly distinct from that of other sounds such as calls, introductory notes, and cage noises. We, therefore, sorted song files (sound files that include at least one full motif of song) and non-song files by focusing on the temporal structure of two acoustic features, sound amplitude and Weiner entropy and by comparing them between a canonical song motif and all sound files as follows. Temporal trajectories of those features were calculated using Sound Analysis Tools for Matlab^60^ for all sound files examined. The canonical song motif was made by averaging amplitude envelopes or entropy trajectories of the most stereotyped part of 10 randomly selected motifs, and the cross-correlation function was calculated between the canonical motif and all sound files; because temporal structure of song motifs varies slightly across renditions, the canonical motif was allowed to undergo ± 10% proportional changes in their temporal pattern. We then plotted the maximum correlation coefficients (mCCs) of amplitude envelopes against mCCs of entropy trajectories for each bird (Supplementary Fig. 3). In these plots, most song files formed a clear cluster around the high mCCs, whereas non-song files were scattered around the low mCCs area. Our preliminary inspections of a subset of files showed that most non-song files had low mCCs in both amplitude envelope and envelope trajectory. Therefore, we set thresholds of mCCs in both features and labeled files with mCCs below the thresholds as non-song files. Finally, we visually inspected spectrograms of non-song files and discarded those that did not include any song motifs; files that included song motifs were put back to the song dataset to be analyzed. The initial singing rate was measured as the mean singing rate over a 30-min period starting at the onset of the first song produced after an LO period (the timing of the 30-min period varied across trials depending on the first song latencies). Instantaneous singing rate was measured by counting the occurrence of song bouts (i.e. the number of song files) over 2-min bins and averaged across trials. Baseline singing rates were measured as mean rates over 1-h periods immediately before the LO periods.

In the experiments with four different LO durations, if songs were not produced during the post LO period, the first song latency was assigned a time of 2 h (120 min) and the initial singing rate was assigned zero. Likewise, in the experiments with drug injections (Fig. 5), if birds did not produce any songs post-LO periods (7-h duration), the first song latency was assigned a time of 7 h (420 min) and the initial singing rate was assigned zero.

### Drug injections

For pharmacological manipulations of dopamine or opioid signaling, dopamine or opioid antagonists or the corresponding vehicle was injected into the pectoral muscle at 30 min prior to the offset of LO periods once a day (see Fig. 5A). Injected drugs and their doses were as follows: the dopamine D1 receptor antagonist R(+)-SCH23390 (Millipore Sigma, D054) dissolved in 0.9% saline (0.2 and 1.0 mg/kg); the dopamine D2 receptor antagonist haloperidol (Millipore Sigma, H1512) stored as stock solution in DMSO at − 20 °C and diluted in 0.9% saline before injection (0.2 and 1.0 mg/kg); the opioid receptor antagonist naloxone hydrochloride dihydrate (Millipore Sigma, N7758) dissolved in 0.9% saline (2 and 10 mg/kg). Doses were selected based on literature in songbirds and chickens^16,33–35^. Four birds were tested with lower doses of all three drugs above; other 4 birds were tested with higher doses of those drugs; other 5 birds were tested with both lower and higher doses of those drugs. For each drug at each dose (and corresponding vehicle), injection was made at least twice in each bird, and the results (first song latencies and initial singing rates after 5-h LO) were averaged across injections; the same number of vehicle injections were made. Multiple injections of the same drug were made with inter-injection intervals > 4 days to prevent possible desensitization to the drug. Each drug-injection day was followed by a washout day with no injections. To assess the effect of drugs on general motor behavior, individual birds were videotaped for 30 min immediately after the offset of 5-h LO periods, and the number of hopping and flying was counted by an observer who was blind to the treatment (drug or vehicle) given to the birds.

### Statistical analysis

To analyze the effect of singing suppression on subsequent singing behavior, we compared first song latencies and initial singing rates between 30-min LO and 5-h LO for each bird using a Wilcoxon signed-rank test (α = 0.05); we used a Wilcoxon signed-rank test for group data as well. We examined the effects of singing suppression, with 4 different durations, on singing behavior using one-way ANOVA. To examine the effects of drug administrations, we compared birds’ behaviors (first song latencies, initial singing rates, and general motor behavior) between those after drug administrations and those after vehicle administrations using a Wilcoxon signed-rank test with a Holm-Bonferroni correction, in which the significance threshold (alpha) for rejecting the null hypothesis varies depending on the p-value based rank of individual comparisons and thus is not determined a priori^61^. All statistical analyses were performed using Matlab (RRID: SCR_001622).

## Supplementary Information

Supplementary Information.

## References

[1] Ryan RM, Deci EL (2000). Intrinsic and extrinsic motivations: Classic definitions and new directions. Contemp. Educ. Psychol..

[2] Parisi GI, Kemker R, Part JL, Kanan C, Wermter S (2019). Continual lifelong learning with neural networks: A review. Neural Netw..

[3] Studer B, Knecht S (2016). Motivation: What have we learned and what is still missing?. Prog. Brain Res..

[4] Di Domenico SI, Ryan RM (2017). The emerging neuroscience of intrinsic motivation: A new frontier in self-determination research. Front. Hum. Neurosci..

[5] Dunn AM, Zann RA (1996). Undirected song in wild zebra finch flocks: Contexts and effects of mate removal. Ethology.

[6] Derégnaucourt S, Mitra PP, Fehér O, Pytte C, Tchernichovski O (2005). How sleep affects the developmental learning of bird song. Nature.

[7] Mori C, Wada K (2015). Audition-independent vocal crystallization associated with intrinsic developmental gene expression dynamics. J. Neurosci. Off. J. Soc. Neurosci..

[8] Ohgushi E, Mori C, Wada K (2015). Diurnal oscillation of vocal development associated with clustered singing by juvenile songbirds. J. Exp. Biol..

[9] Konishi M (1965). The role of auditory feedback in the control of vocalization in the white-crowned sparrow. Z. Für Tierpsychol..

[10] Lombardino AJ, Nottebohm F (2000). Age at deafening affects the stability of learned song in adult male zebra finches. J. Neurosci..

[11] Brainard MS, Doupe AJ (2001). Postlearning consolidation of birdsong: Stabilizing effects of age and anterior forebrain lesions. J. Neurosci..

[12] Tumer EC, Brainard MS (2007). Performance variability enables adaptive plasticity of ‘crystallized’ adult birdsong. Nature.

[13] Riters LV, Stevenson SA (2012). Reward and vocal production: Song-associated place preference in songbirds. Physiol. Behav..

[14] Soderstrom K, Johnson F (2001). Zebra finch CB1 cannabinoid receptor: Pharmacology and in vivo and in vitro effects of activation. J. Pharmacol. Exp. Ther..

[15] Harding CF (2004). Hormonal modulation of singing: Hormonal modulation of the songbird brain and singing behavior. Ann. N. Y. Acad. Sci..

[16] Khurshid N, Jayaprakash N, Hameed LS, Mohanasundaram S, Iyengar S (2010). Opioid modulation of song in male zebra finches (Taenopygia guttata). Behav. Brain Res..

[17] Berridge KC (1996). Food reward: Brain substrates of wanting and liking. Neurosci. Biobehav. Rev..

[18] Berridge KC (2007). The debate over dopamine’s role in reward: The case for incentive salience. Psychopharmacology.

[19] Salamone JD, Correa M (2012). The mysterious motivational functions of mesolimbic dopamine. Neuron.

[20] Fields HL, Margolis EB (2015). Understanding opioid reward. Trends Neurosci..

[21] Sasaki A, Sotnikova TD, Gainetdinov RR, Jarvis ED (2006). Social context-dependent singing-regulated dopamine. J. Neurosci..

[22] Heimovics SA, Cornil CA, Ball GF, Riters LV (2009). D1-like dopamine receptor density in nuclei involved in social behavior correlates with song in a context-dependent fashion in male European starlings. Neuroscience.

[23] Kubikova L, Wada K, Jarvis ED (2010). Dopamine receptors in a songbird brain. J. Comp. Neurol..

[24] Kelm-Nelson CA, Riters LV (2013). Curvilinear relationships between mu-opioid receptor labeling and undirected song in male European starlings (Sturnus vulgaris). Brain Res..

[25] Riters LV, Stevenson SA, DeVries MS, Cordes MA (2014). Reward associated with singing behavior correlates with opioid-related gene expression in the medial preoptic nucleus in male European starlings. PLoS ONE.

[26] Merullo DP, Angyal CS, Stevenson SA, Riters LV (2016). Song in an affiliative context relates to the neural expression of dopamine- and neurotensin-related genes in male European starlings. Brain. Behav. Evol..

[27] Stevenson SA (2020). Endogenous opioids facilitate intrinsically-rewarded birdsong. Sci. Rep..

[28] Volkow ND, Wise RA, Baler R (2017). The dopamine motive system: implications for drug and food addiction. Nat. Rev. Neurosci..

[29] Berke JD (2018). What does dopamine mean?. Nat. Neurosci..

[30] Mohebi A (2019). Dissociable dopamine dynamics for learning and motivation. Nature.

[31] Gil D, Llusia D, Aubin T, Mathevon N (2020). The bird dawn chorus revisited. Coding Strategies in Vertebrate Acoustic Communication.

[32] Hayase S (2018). Vocal practice regulates singing activity–dependent genes underlying age-independent vocal learning in songbirds. PLOS Biol..

[33] Riters LV, Schroeder MB, Auger CJ, Eens M, Pinxten R, Ball GF (2005). Evidence for opioid involvement in the regulation of song production in male European starlings (Sturnus vulgaris). Behav. Neurosci..

[34] Schroeder MB, Riters LV (2006). Pharmacological manipulations of dopamine and opioids have differential effects on sexually motivated song in male European starlings. Physiol. Behav..

[35] Moe RO (2011). Effects of haloperidol, a dopamine D2-like receptor antagonist, on reward-related behaviors in laying hens. Physiol. Behav..

[36] Beaulieu J-M, Gainetdinov RR (2011). The physiology, signaling, and pharmacology of dopamine receptors. Pharmacol. Rev..

[37] Berkowitz BA, Ngai SH, Hempstead J, Spector S (1975). Disposition of naloxone: Use of a new radioimmunoassay. J. Pharmacol. Exp. Ther..

[38] Leonardo A, Konishi M (1999). Decrystallization of adult birdsong by perturbation of auditory feedback. Nature.

[39] Andalman AS, Fee MS (2009). A basal ganglia-forebrain circuit in the songbird biases motor output to avoid vocal errors. Proc. Natl. Acad. Sci. U. S. A..

[40] Charlesworth JD, Tumer EC, Warren TL, Brainard MS (2011). Learning the microstructure of successful behavior. Nat. Neurosci..

[41] Kojima S, Kao MH, Doupe AJ, Brainard MS (2018). The avian basal ganglia are a source of rapid behavioral variation that enables vocal motor exploration. J. Neurosci..

[42] Tachibana RO, Takahasi M, Hessler NA, Okanoya K (2017). Maturation-dependent control of vocal temporal plasticity in a songbird. Dev. Neurobiol..

[43] Lorenz K (1950). The comparative method in studying innate behaviourpatterns. Symp. Soc. Exp. Biol..

[44] Yanagihara S, Hessler NA (2006). Modulation of singing-related activity in the songbird ventral tegmental area by social context. Eur. J. Neurosci..

[45] Goldberg J, Fee M (2011). Vocal babbling in songbirds requires the basal ganglia-recipient motor thalamus but not the basal ganglia. J. Neurophysiol..

[46] Ali F, Otchy TM, Pehlevan C, Fantana AL, Burak Y, Ölveczky BP (2013). The basal ganglia is necessary for learning spectral, but not temporal, features of birdsong. Neuron.

[47] Kojima S, Kao MH, Doupe AJ (2013). Task-related ‘cortical’ bursting depends critically on basal ganglia input and is linked to vocal plasticity. Proc. Natl. Acad. Sci. U. S. A..

[48] Kubikova L, Bosikova E, Cvikova M, Lukacova K, Scharff C, Jarvis ED (2014). Basal ganglia function, stuttering, sequencing, and repair in adult songbirds. Sci. Rep..

[49] Miller JE, Hafzalla GW, Burkett ZD, Fox CM, White SA (2015). Reduced vocal variability in a zebra finch model of dopamine depletion: implications for Parkinson disease. Physiol. Rep..

[50] Hoffmann LA, Saravanan V, Wood AN, He L, Sober SJ (2016). Dopaminergic contributions to vocal learning. J. Neurosci. Off. J. Soc. Neurosci..

[51] Xiao L, Chattree G, Oscos FG, Cao M, Wanat MJ, Roberts TF (2018). A basal ganglia circuit sufficient to guide birdsong learning. Neuron.

[52] Gadagkar V, Puzerey PA, Chen R, Baird-Daniel E, Farhang AR, Goldberg JH (2016). Dopamine neurons encode performance error in singing birds. Science.

[53] Hisey E, Kearney MG, Mooney R (2018). A common neural circuit mechanism for internally guided and externally reinforced forms of motor learning. Nat. Neurosci..

[54] Schultz W, Dayan P, Montague PR (1997). A neural substrate of prediction and reward. Science.

[55] Howe MW, Tierney PL, Sandberg SG, Phillips PEM, Graybiel AM (2013). Prolonged dopamine signalling in striatum signals proximity and value of distant rewards. Nature.

[56] Kuzhikandathil EV, Oxford GS (2002). Classic D1 Dopamine Receptor AntagonistR-(+)-7-Chloro-8-hydroxy-3-methyl-1-phenyl-2,3,4,5-tetrahydro-1*H*-3-benzazepine hydrochloride (SCH23390) Directly Inhibits G Protein-Coupled Inwardly Rectifying Potassium Channels. Mol. Pharmacol..

[57] White RW (1959). Motivation reconsidered: The concept of competence. Psychol. Rev..

[58] Siviy SM (2016). A brain motivated to play: insights into the neurobiology of playfulness. Behaviour.

[59] Vanderschuren LJMJ, Achterberg EJM, Trezza V (2016). The neurobiology of social play and its rewarding value in rats. Neurosci. Biobehav. Rev..

[60] Tchernichovski O, Nottebohm F, Ho C, Pesaran B, Mitra P (2000). A procedure for an automated measurement of song similarity. Anim. Behav..

[61] Holm S (1979). A simple sequentially rejective multiple test procedure. Scand. J. Stat..

