# Supplementary material for: Intrinsic motivation for singing in songbirds is enhanced by temporary singing suppression and regulated by dopamine
Source: Sci Rep. 2021 Oct 13;11:20350. doi: 10.1038/s41598-021-99456-w (PMC8514548; doi:10.1038/s41598-021-99456-w)
Supplement: Supplementary file 1 — Supplementary Information. [file 41598_2021_99456_MOESM1_ESM.docx]

***Supplementary Information***

**Intrinsic motivation for singing in songbirds is enhanced by temporary suppression and regulated by dopamine**

Yunbok Kim^1^, Sojeong Kwon^1^, Raghav Rajan^2^, Chihiro Mori^3^, Satoshi Kojima^1^*

^1^Sensory and Motor Systems Research Group, Korea Brain Research Institute, Daegu, Republic of Korea

^2^Division of Biology, Indian Institute of Science Education and Research Pune, Pune,

Maharashtra, India

^3^ Department of Life Sciences, Graduate School of Arts and Sciences, University of Tokyo, Tokyo, Japan

* Correspondence should be addressed to Satoshi Kojima, Sensory and Motor Systems Research Group, Korea Brain Research Institute, 61, Cheomdan-ro, Dong-gu, Daegu 41068, South Korea. Email: [satoshikojima.sk@gmail.com](mailto:satoshikojima.sk@gmail.com).


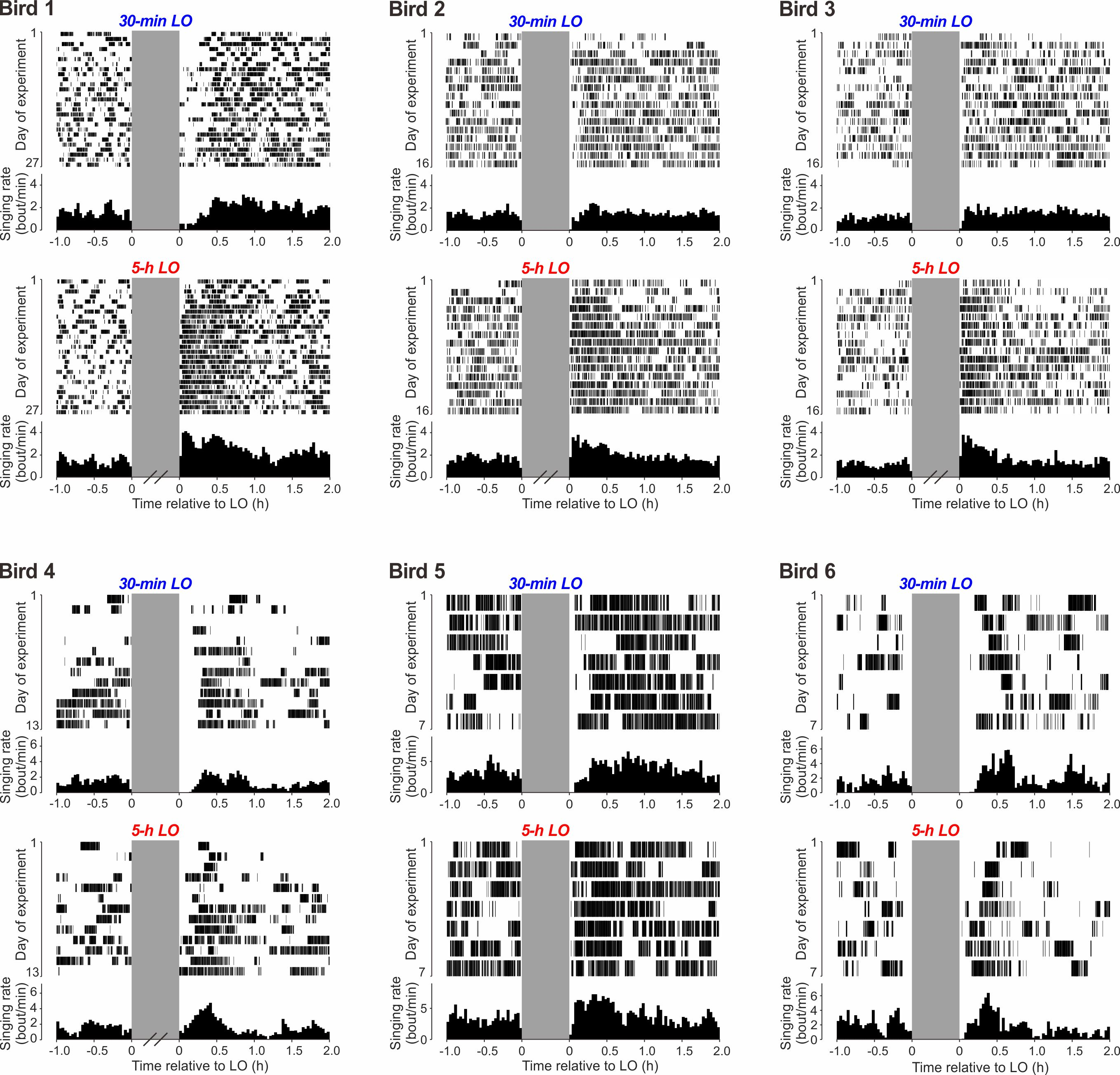


Supplemental Figure 1. Raster plots of song bouts and corresponding singing rate histograms before and after 30-min LO (*top*) and 5-h LO (*bottom*) in 6 young adult birds. Conventions are as in Fig. 1*C*.


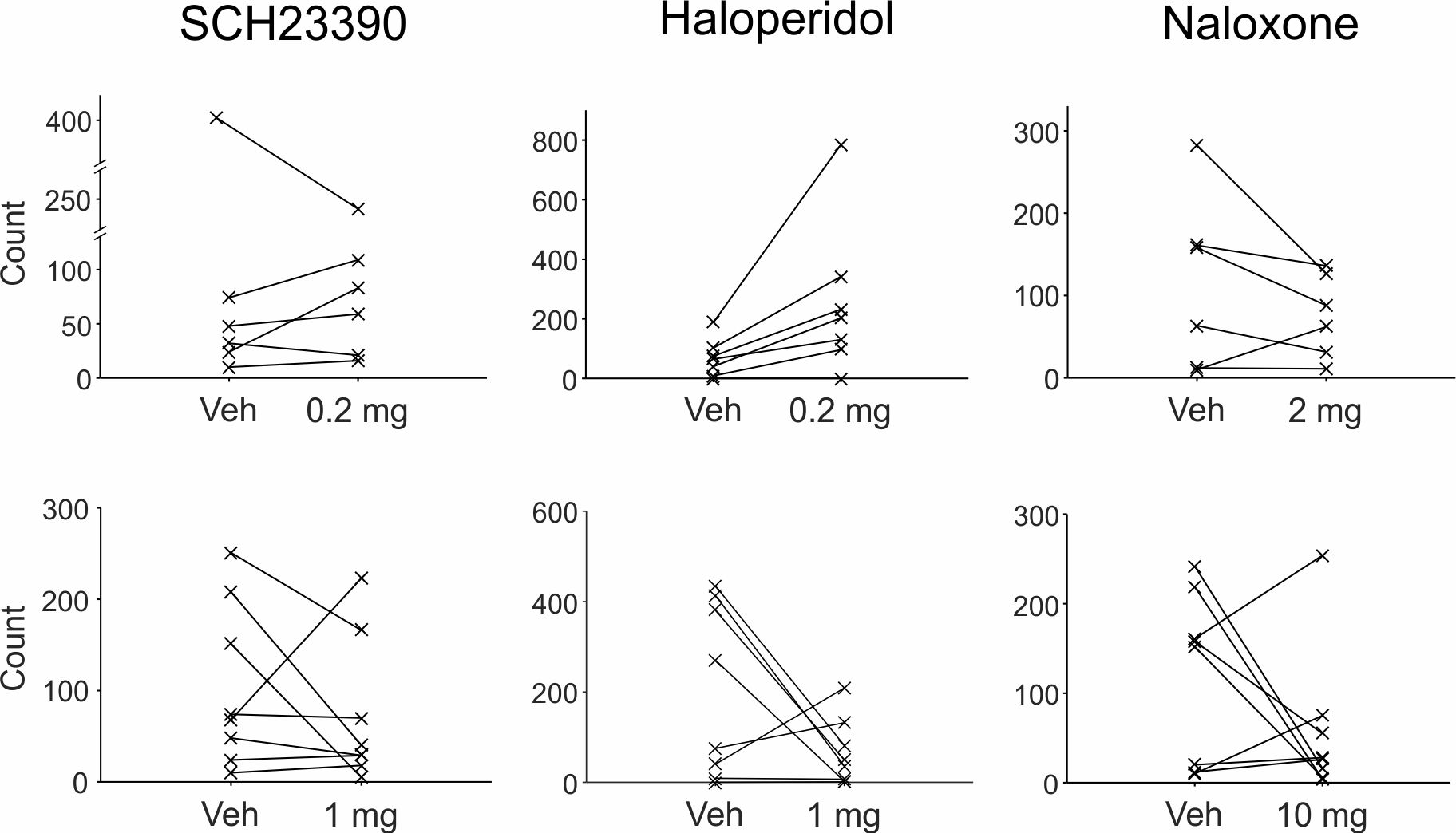


Supplemental Figure 2. Effect of D1 and D2 dopamine receptor antagonists (SCH23390 and haloperidol, respectively) and opioid receptor antagonist (naloxone) on motor behavior (hopping and flying in a cage) measured over a 30-min period following 5-h LO. Conventions are as in Fig. 5B-D. No significant effects were observed for any doses of any drugs examined (*p* = 0.75, 0.46, 0.031, 0.20, 0.22, and 0.31 for 0.2 mg and 1 mg SCH23390, 0.2 mg and 1 mg haloperidol, and 2 mg and 10 mg naloxone, respectively; corrected *α* = 0.0083; Wilcoxon signed-rank test with a Holm-Bonferroni correction for multiple comparisons).


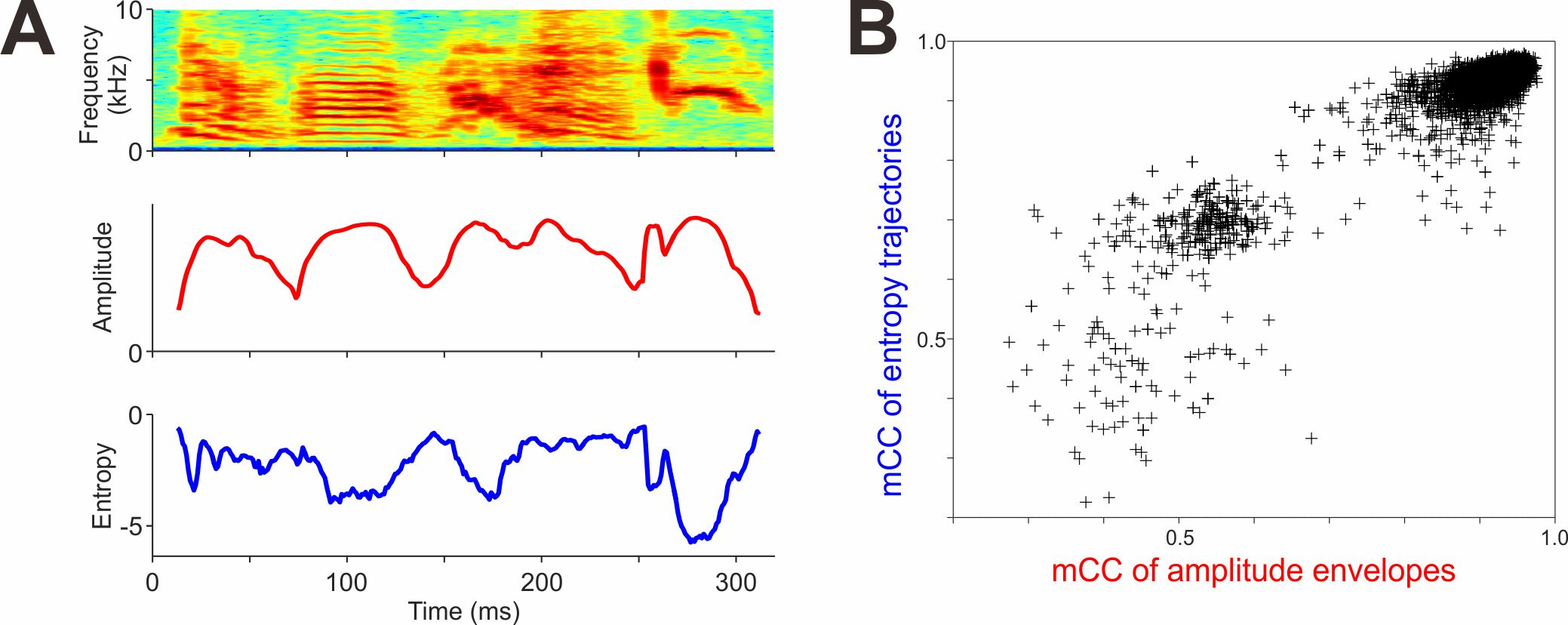


Supplemental Figure 3. Sorting of song files to calculate singing rates. *A.* Examples of a spectrogram (*top*), an amplitude envelope (*middle*), and an entropy trajectory (*bottom*), obtained from a typical song motif. *B.* Scatter plot representing mCCs of amplitude envelope between the canonical motif and sound files and those of entropy trajectory.
